# Case Report: Bilateral Ultrasound-guided Serratus Anterior Plane Blocks for a Chest Wall Burn

**DOI:** 10.5811/cpcem.2020.12.50184

**Published:** 2021-01-29

**Authors:** Tara Benesch, Daniel Mantuani, Arun Nagdev

**Affiliations:** Highland General Hospital, Department of Emergency Medicine, Oakland, California

**Keywords:** ultrasound, serratus anterior plane block, nerve block, burn, pain management

## Abstract

**Introduction:**

The serratus anterior plane block (SAPB) has been shown to effectively treat pain following breast surgery, thoracotomies, and rib fractures. We present the first reported case of a bilateral ultrasound-guided SAPB in a multimodal analgesic regimen after an acute large, thoracic, deep partial-thickness burn.

**Case Report:**

A 72-year-old male presented in severe pain two days after sustaining a deep partial- thickness burn to his anterior chest wall after his shirt caught on fire while cooking. The area of injury was on bilateral chest walls, and the patient was consented for bilateral SAPBs at the level of the third thoracic ribs (T3). With ultrasound guidance, a mixture of ropivacaine and lidocaine with epinephrine was injected into the fascial plane overlying bilateral serratus muscles at T3. The patient reported complete resolution of pain for approximately 15 hours and required minimal additional intravenous analgesia.

**Conclusion:**

The ultrasound-guided SAPB may be an excellent addition to the multimodal analgesic regimen in superficial and partial-thickness burns of the anterior chest wall.

## INTRODUCTION

Chest wall burns are relatively common, and often do not require specialized treatment at a burn center.^1^ In cases of non-circumferential injuries of the chest that do not require surgical intervention, the most common medical management priorities include volume resuscitation and optimal pain management. Mainstays of pain regimens include nonsteroidal anti-inflammatory drugs (NSAIDs), acetaminophen, opioids and ketamine, with ultrasound-guided regional anesthesia uncommonly employed in the non-operative setting.^2^

The ultrasound-guided serratus anterior plane block (SAPB), first described by Blanco et al in 2013, is an effective method of achieving analgesia of the hemithorax from the second thoracic (T2) to ninth thoracic (T9) dermatomes.^3^ Approximately 25–30 milliliters (mL) of anesthetic is injected into the fascial plane either superficial or deep to the serratus anterior (SA) muscle. This targets lateral cutaneous branches of the thoracic intercostal nerves that reside in these fascial planes. SAPBs have been used to reduce pain following breast surgeries, major lung resection, and cardioverter defibrillator insertion.^4^–^7^ More recently, the SAPB is being used in the emergency department (ED) to treat severe pain from rib fractures, herpes zoster, and tube thoracostomy placement.^8^, ^9^ This technique can be easily and safely performed at the bedside and offers another option in the multimodal strategy for pain control in a variety of thoracic injuries. We present the first case of bilateral ultrasound-guided SAPBs incorporated into a multimodal acute pain regimen in a case of large thoracic partial-thickness burn in the ED.

## CASE REPORT

**Image 1:**
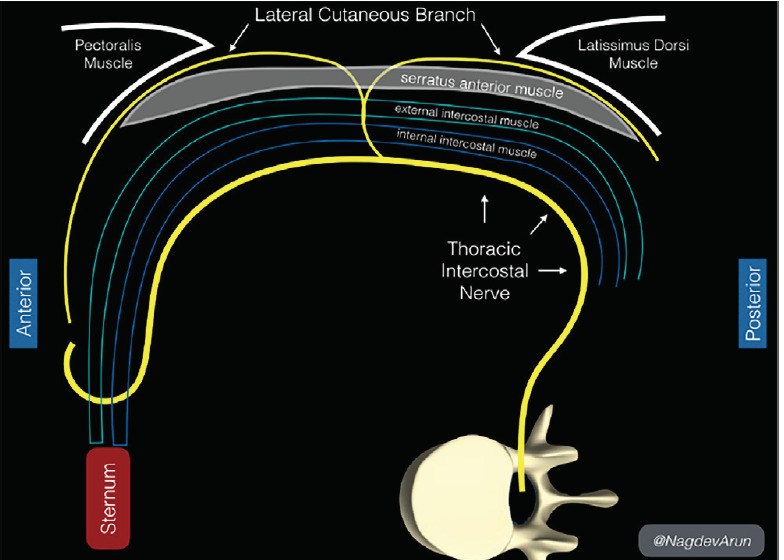
Partial-thickness burn of the anterior chest wall in the patient prior to receiving bilateral serratus anterior plane blocks for pain.

A 72-year-old male with diabetic polyneuropathy presented to the ED with a chief complaint of chest pain two days after sustaining a flame burn while cooking. His vital signs were stable and his physical exam revealed a deep partial-thickness burn over the majority of his right pectoralis extending across midline to the left sternal border (Image 1). The patient reported severe thoracic pain (10/10) unrelieved with oral therapy consisting of NSAIDs and acetaminophen at home.

After obtaining informed consent, bilateral SABPs were performed in the manner described by Blanco et al.^3^ The anesthetic dose was calculated based on the patient’s weight of 66 kilograms (kg) and recommended maximum dose of 3 milligrams (mg)/kg ropivacaine. This yielded a maximum of 20 milliliters (mL) of 1% ropivacaine (10mg/mL) total for both SABPs, or 10 mL per side. An additional 5 mL of 1% lidocaine with epinephrine was added to each SABP to prolong the effect of the block and reduce the systemic absorption of ropivacaine.^10^

**CPC-EM Capsule**

What do we already know about this clinical entity?

Serratus anterior plane blocks (SAPB) effectively treat pain following breast surgery, thoracotomies, and rib fractures.

What makes this presentation of disease reportable?

This is the first description of an ultrasound-guided SAPB performed in the emergency department (ED) for the management of pain from an extensive partial-thickness thoracic burn.

What is the major learning point?

Ultrasound-guided SAPBs can provide safe and rapid analgesia for patients with superficial and partial-thickness chest wall burns in the ED.

How might this improve emergency medicine practice?

Ultrasound-guided SAPBs can facilitate chest wall burn treatments such as dressing changes and wound debridement while reducing reliance on opioid medications.

The patient was positioned in the left lateral decubitus position with right arm overhead for the right-sided block. A high-frequency 10-5 megahertz linear transducer was used to locate the third rib in cross section in the mid-axillary line. Superficial to the rib lies the SA, which is deep to the latissimus dorsi (LD) (Figure). The targeted lateral cutaneous branches of the intercostal nerves at thoracic vertebral levels three to nine (T3–T9), are located in the fascial plane separating the SA and LD muscles. Anesthetic spreads in the fascial plane above and below the level of injection to the lateral cutaneous branches of the thoracic intercostal nerves. Although anesthetic will affect dermatomes above and below the site of injection, it is necessary to adjust the injection site based on the dermatome level. We recommend injecting just above the superior level of injury, targeting the level above the dermatome in case of miscounting the ribs. In this patient, whose burns were located in the T2-T5 dermatomes, we injected at the T2 dermatome above the third rib at the mid-axillary line.

**Image 2:**
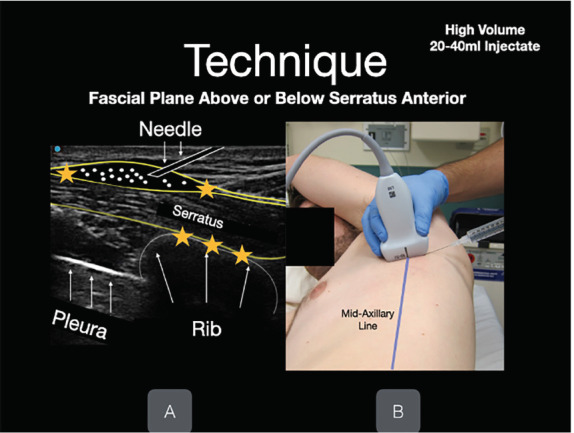
An in-plane technique is used to inject the mixture of anesthetic (in this case, ropivacaine and lidocaine with epinephrine) between serratus anterior and latissimus dorsi muscles. Hydrodissection with 10 milliliters of normal saline prior to injecting anesthetic can assist with visualization of the fascial plane (white dots). The rib and pleura lie beneath the serratus anterior muscle (white arrows). Photo obtained with consent for demonstration purposes.

After sterilizing and anesthetizing the skin, an in-plane technique was used to inject the 15-mL mixture of ropivacaine and lidocaine with epinephrine between SA and LD muscles (Image 2) following hydrodissection with 10 mL of normal saline. The same process was repeated on the patient’s left side. The injected ropivacaine and lidocaine spreads along the fascial plane above and below the site of injection, a process facilitated by hydrodissection with 10mL of normal saline before and after anesthetic administration. Thus, it is not necessary repeat injections at each thoracic vertebral level to achieve analgesia across the chest wall.

Five minutes after the initial injection, the patient reported significant analgesia over his right chest, and 20 minutes after the termination of the procedure he was able to sleep comfortably. The patient reported no discomfort during the injection process and experienced no side effects. A local burn center recommended admission for treatment with silver sulfadiazine and fluid resuscitation; when the patient was interviewed on hospital day two, he reported the SABP took approximately 20–30 minutes to take full effect and lasted for an estimated 15–18 hours. He ultimately required two doses of 2 mg intravenous morphine at approximately 30 hours and 35 hours after his SABP. His only other medications were acetaminophen 500 mg orally every six hours, ibuprofen 600 mg orally every six hours, and home medications gabapentin and trazodone. The patient was discharged in stable condition on hospital day two.

## DISCUSSION

The SAPB, originally used to control pain following breast surgery, is now becoming increasingly popular among emergency physicians to control pain from thoracic injuries in the ED.^3^,^8^ Although the majority of cases in the literature describe the use of the SAPB to relieve pain from rib fractures, other authors have demonstrated its utility in reducing pain from herpes zoster and tube thoracostomy placement.^8^,^9^ To our knowledge, this is the first description of an ultrasound-guided SAPB used for the management of pain from an extensive partial-thickness thorax burn in the ED.

Burns are a leading cause of accidental injury, many of which will not require management at a burn center. Aggressive pain control is necessary in the treatment of these injuries. A multimodal regimen including opioids and ketamine is often required to achieve adequate analgesia for large burns. However, opioids carry risks and side effects of respiratory depression, nausea/vomiting, constipation, and addiction; and ketamine may cause dizziness, dysphoria, and altered mental status.^11^,^12^ As demonstrated by this case, we believe regional anesthesia is a valuable tool in a multimodal pain control regiment for burn patients in the ED. Peripheral nerve blocks have been effectively used for analgesia in patients undergoing wound debridement and dressing changes at burn centers.^13^ A single-injection SAPB can provide approximately 12 hours or more of pain relief to the anterolateral hemithorax without affecting motor function, and may be an ideal option for pain relief prior to dressing changes, wound debridement, or rehabilitation exercises in patients with chest wall burns.

With regard to anesthetic choice, ropivacaine was selected for its long duration of anesthesia and good safety profile. When compared to lidocaine, ropivacaine has a slightly longer mean onset time but provides a much longer mean duration of anesthesia (21.5 hours for ropivacaine compared to 2.4 hours for lidocaine in digital blocks).^14^ Although bupivacaine has a similar mean duration of anesthesia as ropivacaine, ropivacaine has fewer motor effects and a better safety profile (including being safe to use in pregnancy).^15^–^17^

We recognize that the ultrasound-guided SAPB may not be ideal for burns posterior to the mid-axillary line. We have noted minimal success of this planar block in patients with posterior rib fractures and are unclear of the efficacy of this block for these injuries. We also recognize that clinicians performing high-volume SAPB blocks should be aware of maximal anesthetic dosing to prevent administering a toxic dose. The patient’s weight should be measured and a dosing calculator should be used to ensure safety.

## CONCLUSION

Ultrasound-guided serratus anterior plane blocks can potentially provide a safe and rapid method of analgesia for patients with superficial and partial-thickness chest wall burns in the ED. They may be easily performed at the bedside with minimal risk of affecting respiration, mental status, or motor function. As such, they may be a valuable addition to a multimodal pain regimen used in the evaluation and treatment of chest wall burns, and may facilitate treatments such as dressing changes and wound debridement while reducing reliance on opioid medications.

## Figures and Tables

**Figure:**
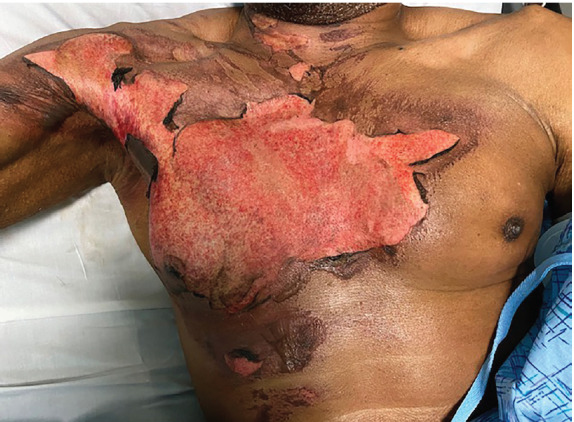
Anatomical landmarks in the serratus anterior plane block. Above the rib lies the serratus anterior (SA), which is deep to the latissimus dorsi (LD). The targeted lateral cutaneous branches of the thoracic intercostal nerves (third to ninth thoracic vertebral level, or T3–T9, arrows), are in the fascial plane separating the SA and LD muscles.
